# Home Alone: Elimination of All but One Alternative Sigma Factor in *Listeria monocytogenes* Allows Prediction of New Roles for σ^B^

**DOI:** 10.3389/fmicb.2017.01910

**Published:** 2017-10-11

**Authors:** Yichang Liu, Renato H. Orsi, Kathryn J. Boor, Martin Wiedmann, Veronica Guariglia-Oropeza

**Affiliations:** Department of Food Science, Cornell University, Ithaca, NY, United States

**Keywords:** RNA-seq, *Listeria monocytogenes*, sigma B, overlapping regulons, promoter

## Abstract

Among *Listeria monocytogenes'* four alternative σ factors, σ^B^ controls the largest regulon. As σ^B^-dependent transcription of some genes may be masked by overlaps among regulons, and as some σ^B^-dependent genes are expressed only under very specific conditions, we hypothesized that the σ^B^ regulon is not yet fully defined. To further extend our understanding of the σ^B^ regulon, we used RNA-seq to identify σ^B^-dependent genes in an *L. monocytogenes* strain that expresses σ^B^ following rhamnose induction, and in which genes encoding the other alternative sigma factors have been deleted. Analysis of RNA-seq data with multiple bioinformatics approaches, including a sliding window method that detects differentially transcribed 5′ untranslated regions (UTRs), identified 105 σ^B^-dependent transcription units (TUs) comprising 201 genes preceded by σ^B^-dependent promoters. Of these 105 TUs, 7 TUs comprising 15 genes had not been identified previously as σ^B^-dependent. An additional 23 genes not reported previously as σ^B^-dependent were identified in 9 previously recognized σ^B^-dependent TUs. Overall, 38 of these 201 genes had not been identified previously as members of the *L. monocytogenes* σ^B^ regulon. These newly identified σ^B^-dependent genes encode proteins annotated as being involved in transcriptional regulation, oxidative and osmotic stress response, and in metabolism of energy, carbon and nucleotides. In total, 18 putative σ^B^-dependent promoters were newly identified. Interestingly, a number of genes previously identified as σ^B^-dependent did not show significant evidence for σ^B^-dependent transcription in our experiments. Based on promoter analyses, a number of these genes showed evidence for co-regulation by σ^B^ and other transcriptional factors, suggesting that some σ^B^-dependent genes require additional transcriptional regulators along with σ^B^ for transcription. Over-expression of a single alternative sigma factor in the absence of all other alternative sigma factors allowed us to: (i) identify new σ^B^-dependent functions in *L. monocytogenes*, such as regulation of genes involved in 1,2-propanediol utilization (LMRG_00594-LMRG_00611) and biosynthesis of pyrimidine nucleotides (LMRG_00978-LMRG_00985); and (ii) identify new σ^B^-dependent genes involved in stress response and pathogenesis functions. These data further support that σ^B^ not only regulates stress response functions, but also plays a broad role in *L. monocytogenes* homeostasis and resilience.

## Introduction

*Listeria monocytogenes* is a Gram-positive foodborne pathogen that causes the serious invasive disease listeriosis, predominantly in susceptible populations such as the immunocompromised, pregnant women, and adults over 65 years old (Goulet et al., 2012). In the US, *L. monocytogenes* causes around 1,600 human listeriosis cases resulting in ~260 deaths annually (Scallan et al., 2011). *L. monocytogenes'* ability to rapidly respond to changing environmental conditions enables it to survive under a wide range of circumstances such as those that may be encountered during food processing as well as in animal or human hosts (Gray et al., 2006). Alternative sigma factors represent one key regulatory mechanism that allows bacteria to adjust rapidly to different environments. Differential association between alternative sigma factors and core RNA polymerase allows the RNA polymerase to recognize specific promoter sequences and initiate transcription of targeted genes under specific conditions. *L. monocytogenes* has up to four alternative sigma factors (σ^B^, σ^C^, σ^H^, and σ^L^) in addition to the housekeeping sigma factor σ^A^. The four alternative sigma factors regulate transcription of genes important for virulence and for response to various stress and growth conditions (Chaturongakul et al., 2011). To date, the general stress response regulator σ^B^ is the most extensively studied alternative sigma factor in *L. monocytogenes*; σ^B^ has been shown to control a regulon of more than 180 genes (Raengpradub et al., 2008). Specifically, σ^B^ plays important roles in virulence and stress response, including transition to stationary phase and resistance to acid, osmotic, arsenate, oxidative, and cold stresses (O'byrne and Karatzas, 2008; Mujahid et al., 2013b).

Regulons controlled by other *L. monocytogenes* alternative sigma factors are less well defined. σ^C^, which is present only in lineage II strains of *L. monocytogenes*, appears to have a small regulon (<10 genes) that contributes to thermal resistance (Zhang et al., 2005). σ^L^, which is a member of the σ^54^ family, has a regulon of >70 genes involved in carbon and amino acid metabolism and cold stress (Raimann et al., 2009). σ^H^ appears to have the second largest regulon among the alternative sigma factors (>150 genes) (Chaturongakul et al., 2011) and has been reported to contribute to growth in minimal media, alkaline stress response, virulence (Rea et al., 2004), and competence (Liu et al., 2016; Medrano Romero and Morikawa, 2016). Previous studies in *L. monocytogenes* have identified considerable overlaps among the regulons controlled by these four alternative sigma factors (Chaturongakul et al., 2011; Mujahid et al., 2013a). Redundant regulation of a gene by multiple sigma factors may make it difficult to classify a gene as a member of a specific regulon following deletion of a single alternative sigma factor. Hence the commonly used approach of comparing transcript levels between a wild type (WT) strain and a corresponding isogenic mutant bearing a deletion of a targeted alternative sigma factor gene may not provide a complete picture of the regulon for a given alternative sigma factor as the approach may fail to identify all genes that are co-regulated by multiple transcriptional regulators. Furthermore, many of the previous transcriptional studies of the σ^B^ regulon (Chatterjee et al., 2006; Ollinger et al., 2008, 2009; Raengpradub et al., 2008; Toledo-Arana et al., 2009; Oliver et al., 2010; Chaturongakul et al., 2011; Ribeiro et al., 2014) have used microarray technology, which is heavily affected by extrinsic noise and is limited to annotated open reading frames (ORFs). In order to provide a more complete definition of the *L. monocytogenes* σ^B^ regulon, we performed RNA sequencing (RNA-seq) using an *L. monocytogenes* strain that expresses σ^B^ under an inducible promoter as the only active alternative sigma factor, thereby removing potential redundancies in regulation by σ^C^, σ^H^, and σ^L^.

## Materials and methods

### Bacterial strains, mutant construction

The quadruple alternative σ factor mutant *(*Δ*BCHL*; FSL C3-0135*)* of *L. monocytogenes* strain 10403S (Mujahid et al., 2013a), which was used here as the background strain, was modified further to overexpress *sigB* from a rhamnose inducible promoter. The strain was constructed using methods analogous to those previously reported for construction of a strain overexpressing σ^H^ (Liu et al., 2016). Briefly, the *sigB* ORF was amplified from *L. monocytogenes* 10403S by PCR and cloned downstream of the rhamnose inducible promoter P_rha_ in plasmid pLF1 (Fieseler et al., 2012); this construct was transformed into *E. coli* strain SM10 λpir to allow for subsequent conjugation of the plasmid into *L. monocytogenes* 10403S Δ*BCHL*, followed by chromosomal integration of the *P*_*rha*_-*sigB* construct in an arginine tRNA gene locus (yielding strain 10403S::Δ*BCHL P*_rha_-*sigB*; FSL B2-0425) (Lauer et al., 2002). A control strain (Δ*BCHL-P*_*rha*_; FSL B2-0429) was constructed by introducing plasmid pLF1 into the chromosome of *L. monocytogenes* 10403S Δ*BCHL* through conjugation and chromosomal integration (see Supplementary Table 1 for strains, plasmids and primers used).

### Growth conditions and rhamnose induction

Strains were streaked from frozen Brain Heart Infusion (BHI) glycerol stocks onto a BHI agar plate, followed by incubation at 37°C for 24 h. A single colony was subsequently inoculated into 5 ml of BHI broth in a 10 ml tube and incubated at 37°C for 18 h with shaking (220 rpm). After incubation, 50 μl BHI culture was inoculated into fresh 5 ml BHI broth in a 10 ml tube and grown to OD_600_ 0.4–0.5 at 37°C with shaking. Induction of *sigB* transcription was performed by adding 250 μl of 1 M rhamnose stock solution to 5 ml OD_600_ 0.4–0.5 bacterial cultures (for a final concentration of 50 mM rhamnose), followed by incubation at 37°C for an additional 30 min. Induction with rhamnose was performed for both 10403S::Δ*BCHL P*_*rha*_-*sigB* and Δ*BCHL-P*_*rha*_. qRT-PCR using the SYBR Green Master Mix Reagent (Life Technologies) and the ABI Prism 7000 Sequence Detection System (Applied Biosystems, Foster City, CA) was used to determine 50 mM rhamnose as the optimal rhamnose concentration for *sigB* induction (Supplementary Figure 1). For these experiments, transcript levels were determined for *sigB* and the housekeeping gene *rpoB* in strain 10403S::Δ*BCHL P*_*rha*_-*sigB*. Expression level differences were determined by the ΔΔCt method using the housekeeping gene *rpoB* as reference gene (Livak and Schmittgen, 2001).

### RNA isolation and sequencing

To define the σ^B^ regulon, strains 10403S::Δ*BCHL P*_*rha*_-*sigB* and Δ*BCHL-P*_*rha*_ were grown to log phase, followed by induction with 50 mM rhamnose for 30 min, as previously described (Liu et al., 2016). RNA isolation and cDNA library construction were also performed as previously described (Liu et al., 2016). All experiments were performed in three biological replicates. Indexed RNA-seq libraries were quantified by digital PCR and sequencing was carried out on a Hiseq 2500 (single-end, 150-bp per read) at the Cornell Core Facility for RNA-sequencing. RNA-seq data are accessible through GEO Series accession number GSE94284.

### RNA-seq alignment, coverage and differential expression analysis

RNA-seq data analysis was performed as previously described (Liu et al., 2016). Briefly, sequence reads were aligned to a 10403S genome using the BWA-mem algorithm (Li and Durbin, 2010) and the data for coverage per base on sense and antisense strands were obtained separately using samtools (Li et al., 2009). Differential expression of genes between the two strains (Δ*BCHL*::*P*_*rha*_ and Δ*BCHL*::*P*_*rha*_-*sigB)* was initially assessed using the Bayseq package for R version 2.2.0 (Hardcastle and Kelly, 2010). Genes were considered differentially expressed (DE) if the FDR (False Discovery Rate) was <0.05 and the Fold Change (FC) was more than 2.0 or <0.5. Genes with significantly higher transcript levels in the Δ*BCHL-P*_*rha*-_*sigB* strain were identified as upregulated by σ^B^ (FC > 2.0) while genes with significantly higher transcript levels in Δ*BCHL-P*_*rha*_ were identified as downregulated (FC < 0.5). A sliding window method described previously (Liu et al., 2016) was also applied to identify significant differential expression of fragments along the chromosome; this method provides more sensitive identification of σ^B^-dependent genes, particularly if genes are preceded by multiple promoters (as described in Raimann et al., 2009 and Liu et al., 2016). Briefly, the 10403S genome was divided into windows of 51 nt (window size) with 25 nt overlap (sliding window) and the RNA-Seq coverage was obtained for each window. Bayseq was then used, as described above, to identify windows with significant differential expression (FDR < 0.05 and FC > 2.0) between the Δ*BCHL*::*Prha-sigB* and the Δ*BCHL*::*Prha* strains. Overlapping windows were considered as one fragment, and fragments mapped to the same transcription unit (TU) were considered to comprise one TU. TUs are defined as all of the genes and/or noncoding RNAs (ncRNAs) that are included in a transcript initiated from a specific promoter. These analyses used an *L. monocytogenes* 10430S genome annotation where TUs were designated based on an RNA-seq analysis of the 10403S WT strain (Oliver et al., 2009); a file with this genome annotation in Genbank format is available from the authors upon request.

### Comparison between previously identified σ^B^-dependent genes and genes identified in this study

To allow us to define newly identified σ^B^-dependent genes, we compiled data on the *L. monocytogenes* σ^B^ regulon from 12 independent studies that used genome-wide transcriptomics and proteomics approaches to compare different *L. monocytogenes* WT strains and corresponding isogenic *sigB* deletion mutants (Wemekamp-Kamphuis et al., 2004; Chatterjee et al., 2006; Abram et al., 2008a,b; Ollinger et al., 2008, 2009; Orsi et al., 2008; Raengpradub et al., 2008; Oliver et al., 2009, 2010; Toledo-Arana et al., 2009; Chaturongakul et al., 2011; Ribeiro et al., 2014). Combined, these studies identified a total of 902 different genes as σ^B^-dependent; only genes that were identified here, but not among these previously reported 902 genes were classified as “newly identified σ^B^-dependent genes”. We also defined a “high confidence” core σ^B^ regulon for *L. monocytogenes* lineage II (which includes strain 10403S, used here), which contained 184 genes that have been reported as σ^B^-dependent in at least three independent experiments among the 12 studies detailed above. This “high confidence” core σ^B^ regulon was used to define the previously identified σ^B^-dependent genes that were not found to be differentially expressed (NDE) in our study reported here. The datasets supporting the conclusions of this article are available in the GEO repository (Private link for reviewers: https://www.ncbi.nlm.nih.gov/geo/query/acc.cgi?token=gbktssowflaften&acc=GSE94284).

### Promoter search and consensus

The transcription start sites (TSS) and 5′ untranslated regions (UTRs) of the differentially expressed TUs were manually annotated based on visual inspection of the locations where RNA-seq coverage data abruptly increased. Subsequently, the 5-50 nt regions upstream of the TSSs were manually scanned for σ^B^-dependent promoter consensus sequences (GTTT-N_12−16_-GGGTAT). A consensus sequence logo of the newly identified σ^B^-dependent promoters was generated using the WebLogo generator (Crooks et al., 2004).

### 5′ rapid amplification of cDNA ends (5′ RACE) analysis

Six newly identified σ^B^-dependent promoters were selected to map promoter regions with the 5′ RACE system (ThermoFisher); these experiments were performed in three biological replicates. Briefly, isolated RNA was reverse transcribed into cDNA with gene-specific primers and cDNA was tailed with dCTP by terminal transferase. The products were then amplified with a nested gene-specific primer and an abridged anchored primer in a touchdown PCR with DreamTaq PCR master mix (ThermoFisher). PCR products were visually analyzed with agarose gel electrophoresis.

### Go term enrichment analysis

Gene Ontology (GO) enrichment analysis to identify GO terms enriched among all 201 genes identified in the 105 σ^B^-dependent TUs was performed using the GOseq 1.24.0 package for R (Young et al., 2010) as previously described (Tang et al., 2015). All GO terms containing more than five genes were analyzed for enrichment. FDR correction for multiple testing was applied and only GO terms with FDR < 0.05 were considered significant.

### Comparative genomics of newly identified σ^B^-dependent promoters

Newly identified σ^B^-dependent promoters were searched against a database containing 27 finished genomes representing *L. monocytogenes* lineage I (*n* = 10), *L. monocytogenes* lineage II (*n* = 9), *L. monocytogenes* lineage III (*n* = 4), *Listeria innocua* (*n* = 1), *Listeria seeligeri* (*n* = 1), *Listeria ivanovii* (*n* = 1), and *Listeria welshimeri* (*n* = 1) using BLAST [32]. Matches with coverage >70% and identity >60% were considered significant and the promoters were, therefore, considered to be present in the respective genome.

## Results

### Combined bioinformatics analyses identify a number of new σ^B^-dependent genes that showed significant differential transcript levels in the respective ORF

Using RNA-seq data from an *L. monocytogenes* strain with deletions in genes encoding all four alternative sigma factors *(*Δ*BCHL*) as well as from a corresponding isogenic strain where *sigB* was re-introduced under rhamnose regulation (Δ*BCHL-P*_*rha*_-*sigB*), we employed two separate bioinformatics approaches to identify σ^B^-dependent genes. These bioinformatics approaches included (i) Bayseq analysis that assessed differential expression using RNA-seq coverage for the full length of all annotated genes (“the traditional Bayseq approach”) and (ii) Bayseq analysis that assessed differential expression using RNA-seq coverage for sliding windows that covered the complete *L. monocytogenes* chromosome (“the sliding window approach”).

With the traditional Bayseq approach, we initially identified 141 genes, including 7 ncRNAs, that showed significantly higher transcripts levels (FDR < 0.05; FC > 2.0) in the *L. monocytogenes* strain overexpressing σ^B^ *(*Δ*BCHL-P*_*rha*_-*sigB)*, as compared to the Δ*BCHL* strain that does not express any alternative sigma factors (Table 1). Among the 141 genes with higher transcript levels, 117 were preceded by upstream σ^B^-dependent promoters. When the sliding window approach was applied, we identified 299 fragments that showed significantly higher transcripts levels (FDR < 0.05; FC > 2.0) in the *L. monocytogenes* strain overexpressing σ^B^. These fragments represented 177 genes, including 7 ncRNAs; 124 of these genes (including all 7 ncRNAs) were preceded by σ^B^-dependent promoters (Supplementary Table 2). Together, these two analyses identified 193 genes as upregulated by σ^B^; 133 of these genes (including 7 noncoding RNAs) were preceded by σ^B^-dependent promoters (Table 1). These 133 genes are located in 93 TUs that each includes a σ^B^-dependent promoter. Overall, 141 of the 193 upregulated genes identified here had been reported as σ^B^-dependent in previous microarray, RNA-seq, or proteomics studies in *L. monocytogenes* (Wemekamp-Kamphuis et al., 2004; Chatterjee et al., 2006; Abram et al., 2008a,b; Ollinger et al., 2008, 2009; Raengpradub et al., 2008; Oliver et al., 2009, 2010; Toledo-Arana et al., 2009; Chaturongakul et al., 2011; Ribeiro et al., 2014). Among the 52 genes newly identified, with these approaches, as upregulated by σ^B^, 16 genes (representing 10 TUs) were preceded by a putative σ^B^-dependent promoter, while 36 did not include an upstream σ^B^-dependent promoter. In the subsequent sections, we will only focus on those genes that showed both evidence for higher transcript levels in the presence of σ^B^ and have an upstream σ^B^-dependent promoter.

**Table 1:** Summary of traditional Bayseq and sliding window analyses results for upregulated genes and TUs.

**Category^a^**	**Bayseq**	**Sliding window**	**Combined^b^**	**New**
**(1) No. of genes identified as** σ^B^**-dependent** (FC > 2.0; FDR < 0.05)	141	177	193	52
(2) No. of genes in (1) that have upstream σ^B^ promoters	117	124	133	16
(3) σ^B^-dependent TUs represented by genes in (2)	86^c^	87^c^	93^c^	4
**(4)** σ^B^**-dependent TUs that were identified by only 5**′**UTR DE fragment and contain upstream** σ^B^ **promoters**	NA^d^	12	12	3
(5) Genes found in the TUs in (4)	NA	16	16	4
(**6) Total number of TUs with both** σ^B^ **promoters and DE fragment in ORF or 5**′**UTR**^e^	NA	NA	105 TUs^c^	7 TUs (represent 15 genes)
(7) Total gene number of σ^B^ regulon members located in the TUs in (6)	NA	NA	201 genes	38 genes (represent 16 TUs)

a *Indented categories indicates groups that represent a subset of the main, non-indented category in bold*.

b *The category “combined” represents the number of genes or TUs that were identified by either the BaySeq or the sliding window approach or both*.

c *LMRG_02094 has two σ^B^-dependent promoters directly upstream of the gene; this gene was counted as two TUs identified by BaySeq and as two TUs identified by the sliding window analysis. This gene was also counted as two TUs in the “combined” column*.

d *NA means “Not Applicable”*.

e *Category 6 represents the combination of categories (3) and (4)*.

With the traditional Bayseq approach, we also identified 18 genes that showed significantly lower transcripts levels (FDR < 0.05; FC < 0.5; see Supplementary Table 3) in the *L. monocytogenes* strain overexpressing σ^B^ *(*Δ*BCHL-P*_*rha*_-*sigB)*, as compared to the Δ*BCHL* strain. Among these 18 downregulated genes, 9 had been reported as downregulated in previous studies (Wemekamp-Kamphuis et al., 2004; Chatterjee et al., 2006; Abram et al., 2008a,b; Ollinger et al., 2008, 2009; Raengpradub et al., 2008; Oliver et al., 2009, 2010; Toledo-Arana et al., 2009; Chaturongakul et al., 2011; Ribeiro et al., 2014). Most of the nine genes newly identified as downregulated by σ^B^ encode transport proteins, including (i) LMRG_01249 and LMRG_01248, which encode subunits of a PTS galactitol transporter; (ii) LMRG_01581, which encodes ArpJ, an amino acid ABC transporter permease; and (iii) LMRG_01595, which encodes an MFS transporter permease. Other downregulated genes include LMRG_01332, which encodes *Listeria* adhesion protein (Lap), an alcohol acetaldehyde dehydrogenase involved in pathogenesis (Jagadeesan et al., 2010); LMRG_00198, encoding a phosphoglycerate mutase; LMRG_01596, encoding a shikimate 5-dehydrogenase, and LMRG_01597 encoding an NADH oxidase. Interestingly, one of the nine newly identified σ^B^-downregulated genes was LMRG_01250, which encodes a putative transcriptional regulator.

### Sliding window analyses of 5′UTR regions identified 12 additional TUs with σ^B^-dependent transcription and upstream σ^B^-dependent promoters in the absence of significant differential transcript levels in the respective ORF

**Figure 1:**
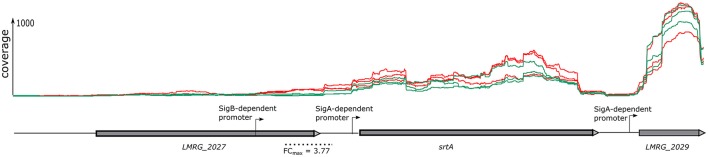
Gene co-transcribed from both σ^B^-dependent and a σ^A^-dependent promoters. RNA-seq coverage is shown for the Δ*BCHL P*_*rha*_-*sigB* strain (red) and Δ*BCHL-P*_*rha*_ strain (green). Transcription start sites (TSS) are indicated by arrows labeled with the regulating sigma factor. The dotted line below the schematic represents the region found to be differentially expressed (FDR < 0.05) with the sliding window approach. The highest window fold change (FC) is shown under the dotted line.

The sliding window approach used here not only identified differentially regulated genes via identification of differentially transcribed fragments that mapped within an ORF (described above), but also identified differentially transcribed fragments that mapped into different 5′UTR, even if the associated downstream genes did not show evidence for differential transcription. This approach thus identified additional TUs (which may include one or multiple genes) as σ^B^-dependent. For example, in the intergenic region upstream of *srtA* we identified a fragment with an FC of 3.77 located upstream of the *srtA* σ^A^-dependent promoter (Figure 1). Visual inspection of the region upstream of this fragment identified a σ^B^-dependent promoter. Hence, using the sliding window approach, we identified *srtA* as a new σ^B^-dependent TU. Overall, this approach identified 12 additional TUs (Supplementary Table 4) with σ^B^-dependent transcription and upstream σ^B^-dependent promoters. For each of these TUs, the associated genes located in the TU did not show significant evidence for higher transcript levels in the strain overexpressing σ^B^.

### Overall analyses reveal a total of 105 σ^B^-dependent TUs that cover 201 genes, including a number of genes not previously identified as part of the σ^B^ regulon

Overall, our analyses identified 105 σ^B^-dependent TUs (i.e., TUs upregulated in the presence of σ^B^) that are preceded by σ^B^-dependent promoters; this includes (i) 93 TUs that showed significant differential transcript levels in one or multiple ORFs that are part of a TU and (ii) 12 TUs that showed significant differential transcript levels in a fragment located in the 5′UTR. Seven of these 105 TUs had not been previously identified as σ^B^-dependent (meaning none of the 15 genes in these seven TUs had been identified as σ^B^-dependent in previous studies (Wemekamp-Kamphuis et al., 2004; Chatterjee et al., 2006; Abram et al., 2008a,b; Ollinger et al., 2008, 2009; Raengpradub et al., 2008; Oliver et al., 2009, 2010; Toledo-Arana et al., 2009; Chaturongakul et al., 2011; Ribeiro et al., 2014). Overall, the 105 σ^B^-dependent TUs include 201 different genes; 38 of these 201 genes had not been previously identified as members of the *L. monocytogenes* σ^B^ regulon (Tables 1, 2). While additional genes were newly identified as σ^B^-dependent through our bioinformatics approaches (see Table 1), not all of these genes met the dual criteria of having both an upstream σ^B^-dependent promoter and differential upregulation in the Δ*BCHL-P*_*rha*_-*sigB* strain.

**Table 2:** Newly identified σ^B^ regulon members grouped by function.

**New σ^B^ regulon member**	**Locus tag for strain EGDe**	**TU**	**Gene product**
**OXIDATIVE STRESS RESPONSE**			
LMRG_00891	lmo1439	LMRG_00891	Manganese superoxide dismutase
LMRG_02644	lmo0222	LMRG_02644-LMRG_02643	Heat shock protein 33
LMRG_02443	lmo0014	*qoxABCD*	A quinol oxidase
**OSMOTIC STRESS RESPONSE**			
LMRG_02775	lmo1701	LMRG_02775-LMRG_02778	An hypothetical protein
LMRG_02115 LMRG_02116	lmo1015 lmo1016	LMRG_02114-LMRG_02116	Permease protein OpuAB, glycine betaine-binding protein Op of glycine betaine ABC transport system,
**PYRIMIDINE BIOSYNTHESIS**			
LMRG_00978-LMRG_00985	lmo1831- lmo1838	LMRG_00978-LMRG_00985	Enzymes in uridine-5′-phosphate biosynthesis
**1,2-PROPANEDIOL UTILIZATION**			
LMRG_00596-00599, 00601,00603-00606, 00608-00611	lmo1153-1156,1160-1163, 1165-1168	LMRG_00594-LMRG_00611	Enzymes that degrade carbon compound 1,2-propanediol
**METABOLISM OF CARBON**			
LMRG_01960 LMRG_01962	lmo2736 lmo2734	LMRG_01960-LMRG_01963	A glycerate kinase and a glycosyl hydrolase
**TRANSCRIPTIONAL REGULATION**			
LMRG_01913	lmo2784	LMRG_01913	Transcriptional antiterminator of lichenan operon, BglG family
LMRG_00050 LMRG_00051	lmo0359 lmo0360	LMRG_00050-LMRG_00051	A fructose-biphosphate hydrolase and a DeoR family putative transcriptional regulator
**NO CLEARLY ANNOTATED FUNCTIONS**			
LMRG_01659	lmo2173	LMRG_01658-LMRG_01659	σ^L^ -dependent activator
LMRG_01784	lmo2464	LMRG_01784-LMRG_01786	Transcriptional regulator of tetR Family
LMRG_01676	lmo2156	LMRG_01676	A hypothetic protein
LMRG_01199_as	N/A	LMRG_01199_as	Antisense RNA
LMRG_00529	lmo1067	LMRG_00529	GTP-binding protein TypA/BipA
LMRG_02699	lmo2569	LMRG_02695-LMRG_02699	Periplasmic oligopeptide-binding protein oppA

### Newly identified σ^B^ regulon members include genes involved in stress response, metabolism of carbon and nucleotides, and virulence

The 38 genes newly identified as members of the σ^B^ regulon (Table 2) encode proteins involved in oxidative and osmotic stress response, metabolism of energy, carbon and nucleotides, transcriptional regulation, and other functions of *L. monocytogenes*. Newly identified σ^B^-dependent genes involved in oxidative stress response include LMRG_00891, LMRG_02644, and LMRG_02443, which is the second gene in the *qoxABCD* operon. Our data newly identified σ^B^-dependent promoters upstream of all of these operons. We also newly identified LMRG_02775 as a member of the σ^B^ regulon. While the other members of the LMRG_02775-LMRG_02778 operon had previously been identified as σ^B^-dependent (Oliver et al., 2010; Chaturongakul et al., 2011), we newly identified a σ^B^-dependent promoter upstream of this operon, suggesting direct σ^B^-dependent transcription of this 4-gene operon, which encodes four proteins, including one OsmC/Ohr family protein. While the functions of the genes in this operon have not yet been elucidated, OsmC proteins are induced by ethanol and osmotic stresses and Ohr proteins are induced by organic peroxide and are involved in organic hydroperoxide detoxification in Gram-negative bacteria (Atichartpongkul et al., 2001). In other intracellular pathogens such as *Mycobacterium tuberculosis*, OsmC proteins have been reported to protect against organic hydroperoxide stress (Saikolappan et al., 2011), suggesting this operon may contribute to oxidative or osmotic stress response in *L. monocytogenes*. We also newly identified two members of the LMRG_02114-LMRG_02116 operon as part of the σ^B^ regulon and newly identified a σ^B^-dependent promoter upstream of this operon. This operon encodes the OpuAA, OpuAB, and OpuAC subunits of the glycine betaine ABC transport system. Expression of the *opuA* operon is under osmotic control in *B. subtilis*, and a *B. subtilis* mutant strain lacking the OpuA transport system showed a considerably decreased ability to uptake the osmoprotectant glycine betaine (Kempf and Bremer, 1995).

**Figure 2:**
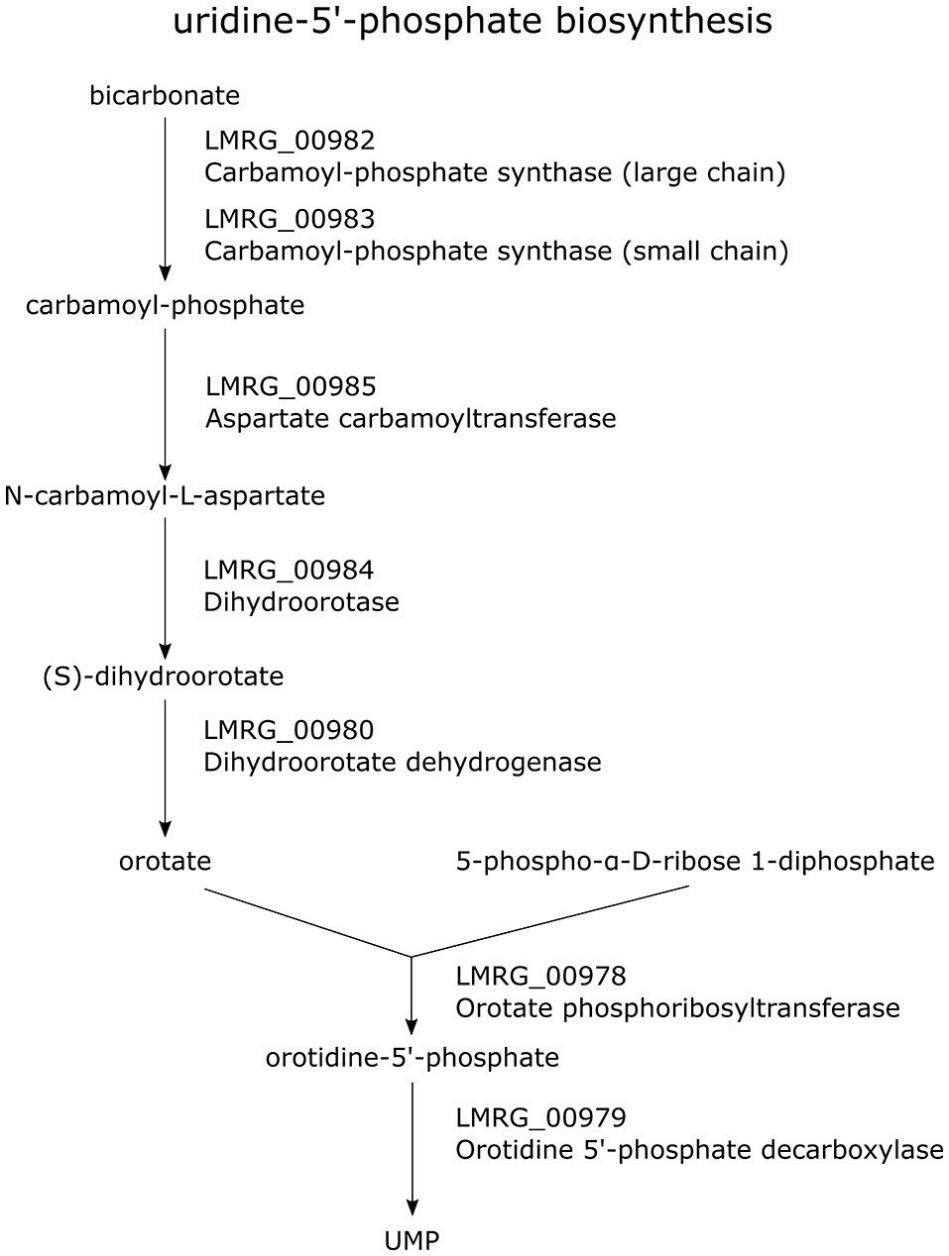
Pathway of uridine-5′-phosphate biosynthesis. Genes encoding the enzymes involved in this pathway are labeled in purple. All genes in the TU LMRG_00985-LMRG_00978 except LMRG_00981 are shown in this pathway.

Our study also identified a number of new members of the σ^B^ regulon involved in energy, carbon and nucleotide metabolism. Specifically, we newly identified LMRG_00978-LMRG_00985 as a σ^B^-dependent TU; genes in this TU encode a set of enzymes involved in uridine-5′-phosphate biosynthesis, a pyrimidine ribonucleotides *de novo* biosynthesis pathway (Figure 2). Importantly, our data also indicate that all genes in the LMRG_00594-LMRG_00611 operon are part of the σ^B^ regulon; this operon encodes all enzymes involved in utilization of carbon compound 1,2-propanediol, which is an important carbon source during infection for bacterial pathogens such as *S. enterica* (Bobik et al., 1999) and was reported to be degraded by *L. innocua* (Xue et al., 2008). In addition to providing direct RNA-seq evidence for significant differential transcript levels for 6 genes in this operon, which had not been previously identified as σ^B^-dependent, we also identified a σ^B^-dependent promoter upstream of this operon; in previous studies, only 5 genes in this 18-gene operon had been identified as σ^B^-dependent (Wemekamp-Kamphuis et al., 2004; Chatterjee et al., 2006; Abram et al., 2008a,b; Ollinger et al., 2008, 2009; Raengpradub et al., 2008; Oliver et al., 2009, 2010; Toledo-Arana et al., 2009; Chaturongakul et al., 2011; Ribeiro et al., 2014). Our data also newly identified a σ^B^-dependent promoter upstream of the three-gene operon LMRG_01960-LMRG_01962 with LMRG_01960 and LMRG_01962 newly identified as σ^B^-dependent. We also newly identified a σ^B^-dependent promoter upstream of LMRG_01432-LMRG_01431, suggesting direct σ^B^ regulation of this operon.

We also identified new members of the σ^B^ regulon that encode transcriptional regulators, further supporting that σ^B^ is part of a complex regulatory network in *L. monocytogenes*. Specifically, we newly identified LMRG_01913, which encodes a lichenan operon transcriptional antiterminator as a σ^B^-dependent TU. In addition, we newly identified LMRG_00050-LMRG_00051 as a σ^B^-dependent TU, in which LMRG_00051 encodes a putative transcriptional regulator. LMRG_01659, encoding a σ^54^-dependent activator (Arous et al., 2004) and LMRG_01784, encoding a TetR family transcriptional regulator, were also newly identified as members of the σ^B^ regulon. While the other genes in TUs LMRG_01658-LMRG_01659 and LMRG_01784-LMRG_01786 had previously been reported as σ^B^-dependent (Raengpradub et al., 2008; Oliver et al., 2009, 2010; Ollinger et al., 2009; Toledo-Arana et al., 2009; Chaturongakul et al., 2011; Ribeiro et al., 2014), we newly identified σ^B^-dependent promoters upstream of both operons.

Our data also identified a σ^B^-dependent promoter upstream of a TU including a single gene encoding sortase A, *srtA*. SrtA is involved in proteolysis and virulence in *L. monocytogenes* (Garandeau et al., 2002). Additional newly identified σ^B^-dependent TUs (where none of the genes in a TU had previously been identified as σ^B^-dependent) included LMRG_00529 and two TUs (LMRG_01676 and LMRG_01199_as) with no clearly annotated functions.

### Gene ontology analyses identify a role for σ^B^ in regulating genes encoding functions involved in transport, homeostasis, pathogenesis, and nucleotide metabolism

Gene ontology (GO) enrichment analysis performed for the σ^B^ regulon (i.e., all 201 genes that were part of the 105 σ^B^-dependent TUs identified here) found 18 GO terms to be overrepresented (see Table 3), including GO terms related to transport, homeostasis, metabolic and catabolic processes. In addition, GO terms related to “pyrimidine nucleotide biosynthetic and metabolic processes”; “glycolysis, metabolic and catabolic processes of propanediol, diol and alcohol,” were also over-represented, suggesting a role for σ^B^ in regulating general metabolic functions.

**Table 3:** Enriched GO terms for 201 genes in 105 σ^B^-dependent TUs^a^.

**GO ID**	**DE genes in this GO term (LMRG_)**	**GO term**
GO:0006066	00050, 00236,00272, 00594, 00600-00607, 00611, 01432, 01627, 01789-01793, 01962, 02219, 02632, 02695	Alcohol metabolic process
GO:0006091	00050, 00981, 01627, 01790-01793, 02442-02444, 02632	Generation of precursor metabolites and energy
GO:0006096	00050, 01627, 01790-01793, 02632	Glycolytic process
GO:0006220	00978-00985, 01076	Pyrimidine nucleotide metabolic process
GO:0006221	00978-00985, 01076	Pyrimidine nucleotide biosynthetic process
GO:0009056	00050, 00221, 00600-00603, 00605, 00607, 00611, 01030, 01627, 01790-01793, 02219, 02304, 02472, 02632	Catabolic process
GO:0015418	00873, 00874, 00877-00880, 02114-02116	Quaternary-ammonium-compound-transporting ATPase activity
GO:0015695	00873, 00874, 02114-02116	Organic cation transport
GO:0015697	00873, 00874, 02114-02116	Quaternary ammonium group transport
GO:0030104	00873, 00874, 02114-02116	Water homeostasis
GO:0034311	00594, 00600-00603, 00605, 00607, 00611	Diol metabolic process
GO:0034313	00600-00603, 00605, 00607, 00611	Diol catabolic process
GO:0044248	00050, 00221, 00600-00603, 00605, 00607, 00611, 01030, 01627, 01790-01793, 02219, 02304, 02472, 02632	Cellular catabolic process
GO:0044275	00050, 00221, 01030, 01627, 01790-01793, 02219, 02632	Cellular carbohydrate catabolic process
GO:0046164	00050, 00600-00607, 00611, 01627, 01790-01793, 02219, 02632	Alcohol catabolic process
GO:0048878	00873, 00874, 02041, 02114-02116	Chemical homeostasis
GO:0051143	00594, 00600-00603, 00605, 00607, 00611	Propanediol metabolic process
GO:0051144	00600-00603, 00605, 00607, 00611	Propanediol catabolic process

a *σ^B^ regulon members are defined as genes in the σ^B^-dependent TUs that are preceded by σ^B^-dependent promoters and showed significant differential transcript levels in one or more fragments located within the TU*.

### Conservation of newly identified σ^B^-dependent promoters in *L. monocytogenes* and *Listeria* spp.

**Figure 3:**
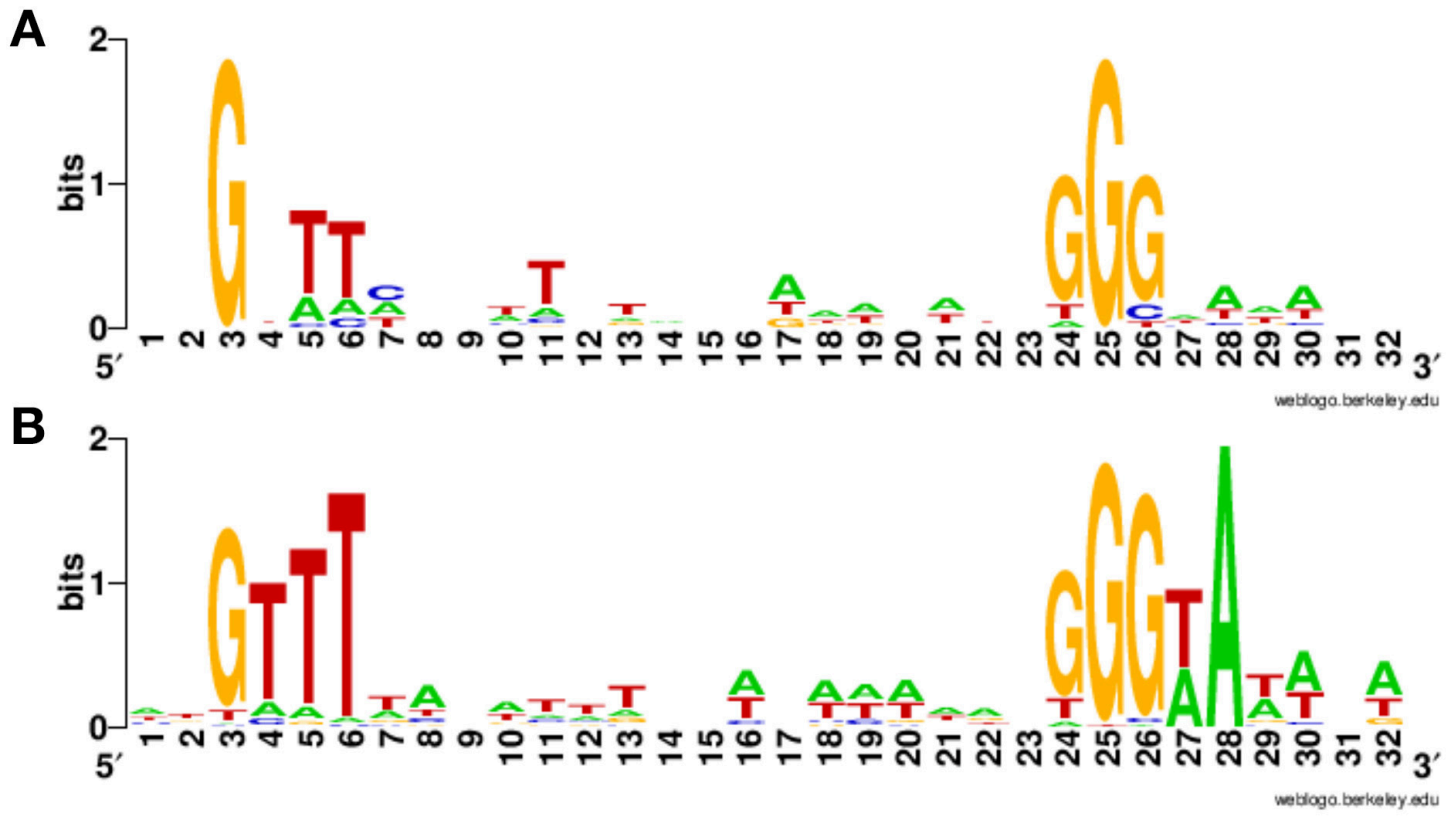
Sequence logo for newly identified putative σ^B^-dependent promoters. **(A)** Sequence logo for the 18 σ^B^-dependent promoters associated with newly identified σ^B^-dependent genes. **(B)** Sequence logo for σ^B^-dependent promoters published in previous study (Oliver et al., 2009).

**Figure 4:**
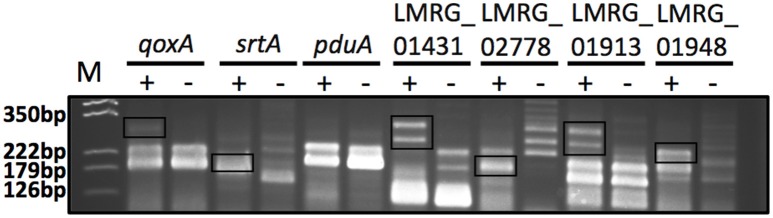
5′RACE PCR confirmation of six putative σB-dependent promoters newly identified here by RNA-seq. The six putative σ^B^-dependent promoters selected for confirmation are located upstream of *qoxA, srtA, pduA*, LMRG_01431, LMRG_02778, and LMRG_01913. LMRG_01948 was used as a positive control, as it was reported to have a strong σ^B^-dependent promoter (Abram et al., 2008b). The image shows the 5′RACE PCR products in a 3% agarose gel; the two lanes for each target represent reaction performed with RNA isolated from the Δ*BCHL P*_*rha*_-*sigB* strain, which expresses σ^B^ (+) and the Δ*BCHL-P*_*rha*_ strain, which does not express σ^B^ (−). The σ^B^-dependent transcript bands are shown in rectangles. Multiple 5′RACE bands were expected for these genes as they were all initially characterized by having a putative σ^B^-dependent promoter as well as an additional σ^A^-dependent promoter. Five of six promoters selected for confirmation displayed clear σ^B^-dependent transcript bands, supporting the existence of σ^B^-dependent promoters. 5′RACE PCR for *pduA* did not yield a band that confirmed the putative σ^B^-dependent promoter that was identified by RNA-seq; a putative σ^A^-dependent promoter was found 14 nt upstream of the putative σ^B^-dependent promoter; transcripts from this σ^A^-dependent promoter may have masked the σ^B^-dependent transcript. The expected sizes of the σ^B^-dependent transcript bands are around 250 bp for *qoxA*, 170 bp for *srtA*, 165 bp for *pduA*, 275 bp for LMRG_01431, 185 bp for LMRG_02778, 275 bp for LMRG_01913, and 210 bp for LMRG_01948. The image was adjusted for contrast, intensity levels and saturation using Photoshop; manipulations were performed on the whole picture and no specific bands were enhanced or modified. The results shown here are representative of three biological replicates.

Overall, we newly identified 18 putative σ^B^-dependent promoters here (see Figure 3 for a consensus sequence). 5′ RACE performed on six of these promoters confirmed five of these promoters as σ^B^-dependent (Figure 4). For *pduA*, where 5′RACE did not detect a clear σ^B^-dependent transcript, a putative σ^A^-dependent promoter was found 14 nt upstream of the putative σ^B^-dependent promoter; transcripts from this σ^A^-dependent promoter may have masked the σ^B^-dependent transcript. Across all 18 newly identified putative σ^B^-dependent promoters, the first three nucleotides of the −10 signal sequence (GGG) are generally conserved among both these newly identified putative σ^B^-dependent promoters and the previously published σ^B^-dependent promoter sequences (Oliver et al., 2009), the last three nucleotides appear to be less conserved among the new promoters identified here, as compared to the previously reported σ^B^-dependent promoter sequences (Figure 3). Through comparative genomics, we assessed that all but one of the 18 σ^B^-dependent promoters newly identified in *L. monocytogenes* 10403S were present in the 23 additional *L. monocytogenes* genomes analyzed here; the σ^B^-dependent promoter upstream of the anti-sense RNA (asRNA), which overlaps LMRG_00359 in the opposite strand, was not found in the four lineage III genomes analyzed (Table 4). Overall, −35 and −10 promoter regions were highly conserved among different strains and lineages, even though some promoters show lineage- or strain-specific sequence features (Table 4). Further analysis showed that only 10 of the 18 σ^B^-dependent promoters newly identified in *L. monocytogenes* were found in all 5 *Listeria* species analyzed (i.e., *L. monocytogenes, L. innocua, L. ivanovii, L. welshimeri*, and *L. seeligeri*) (Table 4). While these comparisons suggest that we identified new σ^B^-dependent functions that are largely conserved in *L. monocytogenes* and to a lesser extent in other *Listeria sensu strictu* species, future comparative genomics analyses that utilize additional closed genomes as they become available will help to further define lineage-, strain-, and species-specific members of the SigB regulon.

**Table 4:** σ^B^-dependent −35 and −10 promoter regions for the newly identified σ^B^-dependent operons.

**Promoters**	***L. monocytogenes***			***L. innocua***	***L. welshimeri***	***L. ivanovii***	***L. seeligeri***
	**Lineage I**	**Lineage II**	**Lineage III**				
**LMRG_00986**							
−35 signal	GCAC	GCAC	GCAC	GCAC	GCAC	ACAC	GCAC
−10 signal	GGCTCT	GGCTCT	GGCTCT	GGCTCT	GGCTCT	GGCTCG	GGCTCT
**LMRG_02643**							
−35 signal	GAAT	GAAT	GAAT	GAAT	GAAT	GAAT	GAAT
−10 signal	GGGCTT	GGGCTT	GGGCTT	GGGCTT	GGGCTT	GGGTTA	GGGTTA
***gbuA***							
−35 signal	GCTT	GCTT^a^	GCTT^a^	GCTT	GCTT	GCTT	GCTT
−10 signal	GGGCCG	GGGCCG	GGGCCG	GGGCCG	GGGCCG	GGGCCG	GGGCCG
**LMRG_00529**							
−35 signal	CGCT	GGCT	CGCT	CGCT	CGCT	CGCC	CGCT
−10 signal	GGTAAG	GGTAAG	GGTAAG	GGTAAG	GGTAAG	GGTAAG	GGTAAG
***pduA***							
−35 signal	GTAA	GTAA	GTAA	GTAA	GTAA	GTAA	GTAA
−10 signal	AGGAGG	AGGAGG	AGGAGG	AGGAGG	AGGAGG	AGGAGG	AGGAGG
**LMRG_02813**							
−35 signal	GTAC	GTAC	GTAC	GTAC	NF^b^	NF	NF
−10 signal	GGGTAT	GGGTAT	GGGTAT	GGGTAT	NF	NF	NF
**LMRG_01676**							
−35 signal	GATT	GATT	AATT	GATA	TATT	GATT	GATT
−10 signal	GGGATA	GGGATA	GGGATA	AGGATA	AGGAAA	AGGATA	AGGATA
***qoxA***							
−35 signal	GTTT^c^	GTTT	GTTT	GTTT	GTTT	GTTT	GTTT
−10 signal	GGGAAT	GGGAAT	GGGAAT	GGGAAT	GGGAAT	GGGAAT	GGGAAT
**LMRG_00050**							
−35 signal	GATA	GATA	GATA	GATA	GATA	NF	NF
−10 signal	TGGTTC	TGGTTC	TGGTTC	TGGTTC	TGGTCC	NF	NF
**LMRG_00271**							
−35 signal	GTTT	GTTT	GTTT	GTTT	GTTT	NF	NF
−10 signal	GGGGAA	GGGGAA	GGGGAA	GGGGAA	GGGGAA	NF	NF
**asRNA(LMRG_00359)**							
−35 signal	GTTT	GTTT	NF	GTTT	NF	NF	NF
−10 signal	GGGCTA	GGGCTA	NF	GAGCTA	NF	NF	NF
***srtA***							
−35 signal	GATT	GATT	GATT	GATT	GATT	GATT	GATT
−10 signal	GGGAAA	GGGAAA	GGGAAA	GGGAAA	GGGAAA	GGGAAA	GGGAAA
**LMRG_02094**							
−35 signal	GGTT	GGTT	GGTT	GGTT	GGTT	GATT	GGTT
−10 signal	GGGTAA	GGGTAA	GGGTAA	GGGTAA	GGGTAA	GGGTAA	GGGAAA
**LMRG_01431**							
−35 signal	GTTA	GTTA	GTTA	GTTA	GTTA	NF	NF
−10 signal	GGGTAA	GGGTAA	GGGTAA	GGGTAA	GGGTAA	NF	NF
**LMRG_02778**							
−35 signal	GCTT	GCTT	GCTT	GCTT	GCTT	GCTT	GCTT
−10 signal	GGGTAT	GGGTAT	GGGTAT	GGGTAT	GAGTAT	GCGTAT	GCGTAT
**asRNA (LMRG_01199)**							
−35 signal	GATT	GATT	GATT	GTTT	NF	ATTT	NF
−10 signal	TGGAAT	TGGAAT	TGGAAT	TGGAGT	NF	TGGAAT	NF
**LMRG_01963**							
−35 signal	GTTT	GTTT	GTTT	NF	NF	NF	NF
−10 signal	AGCAAT	GGCAAT	GGCAAT	NF	NF	NF	NF
**LMRG_01913**							
−35 signal	GTTT	GTTT	GTTT	GTTT	GTTT	GTTT	NF
−10 signal	GG(G/A)CTA^d^	GGGCTA	(T/G)AGCTT^e^	GGGCGA	GGGCGA	GAATTA	NF

a *With the exception of strains 10403S and SLCC5850 (both lineage II strains), all the lineage II and III strains present a deletion of a thymine between the −35 and −10 regions*.

b *NF, Not found*.

c *Strain SLCC2482 (lineage I) presents an insertion of a thymine between the −35 and −10 region*.

d *Strain ATCC has the sequence GGACTA at the −10 region while all other Lineage I strains have the sequence GGGCTA*.

e *Strain SLCC2376 has the sequence GAGCTT at the −10 region while all other lineage III strains have the sequence TAGCTT*.

### Comparison with previously reported σ^B^-dependent regulon members identifies a number of σ^B^-dependent genes that are also regulated by σ^A^

In order to compare the σ^B^-dependent genes identified here to previously reported σ^B^-dependent genes, we used data from previous microarray, RNA-seq and proteomic studies in different strains and under different stress conditions (Wemekamp-Kamphuis et al., 2004; Chatterjee et al., 2006; Abram et al., 2008a,b; Ollinger et al., 2008, 2009; Raengpradub et al., 2008; Oliver et al., 2009, 2010; Toledo-Arana et al., 2009; Chaturongakul et al., 2011; Ribeiro et al., 2014), to define a core σ^B^ regulon for *L. monocytogenes* lineage II, which includes the strain we used here (10403S). Overall, 184 genes have been reported as σ^B^-dependent at least three times in prior publications (representing high confidence for each as a member of the σ^B^ regulon). Our data showed that 110 of the 193 genes that were identified as significantly upregulated by σ^B^ in our study also were included in this “high confidence regulon” of 184 genes. Therefore, overexpression of σ^B^ in Δ*BCHL* failed to identify 74 genes previously reported as upregulated by σ^B^ (Supplementary Table 5).

Promoter searches upstream of the 5′ UTR of these 74 genes identified upstream σ^B^-dependent promoters for 35 of these genes; these 35 genes represent 28 TUs with corresponding σ^B^-dependent promoters. Twenty-three of these 28 TUs had both σ^A^- and σ^B^-dependent promoters (Table 5). Therefore, it is possible that expression from the σ^A^-dependent promoter masked expression from the σ^B^-dependent promoter in these genes, not allowing their identification as σ^B^-dependent in our study. While no clear σ^A^-dependent promoters were identified for the other five TUs, RNA-seq coverage patterns in the quadruple mutant, which only expresses σ^A^, suggested that four of these TUs may be co-regulated by σ^A^. These four TUs were LMRG_00261-LMRG_00262, LMRG_00335-LMRG_00338, LMRG_01794, and *htrA* (LMRG_02579) whose σ^B^-dependent transcription generates a long (473 nt) 5'UTR, which may have a regulatory function.

**Table 5:** Co-regulation of σ^A^ and σ^B^ among the NDE σ^B^-dependent genes.

**TU**	**Genes**	**Other genes in the same operon**	**Promoter identified (from upstream to downstream)**	**Genes or fragments^a^ identified as differentially expressed in this study**	**Comments**
1	LMRG_00100, LMRG_00101	–	σ^B^, σ^A^, σ^A^	No	–
2	LMRG_00262	LMRG_00261	σ^B^	LMRG_00261	σ^A^-dependent expression pattern
3	LMRG_00334	–	σ^B^, σ^A^	No	–
4	LMRG_00336	LMRG_00335, LMRG_00337, LMRG_00338,	σ^B^	LMRG_00335, LMRG_00337,	σ^A^-dependent expression pattern
5	LMRG_00530	–	σ^A^, σ^B^	Frag 43108, Frag 43109	Two DE fragments identified between the promoters
6	LMRG_00672	LMRG_00671	σ^A^, σ^B^	No	–
7	LMRG_00745	–	σ^A^, σ^B^	No	Host factor-I protein
8	LMRG_00884	LMRG_00885	σ^B^, σ^A^	No	–
9	LMRG_00906	LMRG_00910, LMRG_00909, LMRG_00908, LMRG_00907	σ^A^, σ^H^, σ^B^	No	RpoD (σ^A^)
10	LMRG_01076, LMRG_01077	LMRG_01078, LMRG_01079, LMRG_01080	σ^A^, σ^B^	LMRG_01078, LMRG_01079, LMRG_01080	–
11	LMRG_01140	–	σ^A^, σ^B^	No	Alpha-acetotactate decarboxylase
12	LMRG_01284	–	σ^B^, σ^A^	Frag 86921 to 86928	DE fragments identified in 5'UTR
13	LMRG_01361	LMRG_01360	σ^A^, σ^A^, σ^B^	LMRG_01360	–
14	LMRG_01432	LMRG_01431	σ^B^, σ^A^	LMRG_01431	Glycerol kinase
15	LMRG_01737	–	σ^B^, σ^A^	Fragments 101803 to 101809	DE fragments identified in 5'UTR
16	LMRG_01794	–	σ^B^	No	σ^A^-dependent transcription initiating from LMRG_01793 (*eno*) is running through LMRG_01794 despite the presence of a Rho-independent transcription terminator in between
17	LMRG_02000, LMRG_02001, LMRG_02002	–	σ^A^, σ^B^	No	–
18	LMRG_02028	–	σ^A^, σ^B^		*srtA*, DE fragments identified in 5'UTR
19	LMRG_02055, LMRG_02056	LMRG_02057	σ^B^, σ^A^	frag 38333	*nagA* and *nagB* operon. Two NagR attenuatormotives were identified suggesting co-regulation with NagR
20	LMRG_02215, LMRG_02216	LMRG_02217	σ^A^, σ^B^	LMRG_02217	–
21	LMRG_02219	LMRG_02218	σ^B^, σ^A^	LMRG_02218	*rpiB*
22	LMRG_02317, LMRG_02320	LMRG_02311, LMRG_02312, LMRG_02313, LMRG_02314, LMRG_02315, LMRG_02316, LMRG_02318, LMRG_02319	σ^A^, σ^B^		*rsbV* and *rsbX* from *sigB* operon
23	LMRG_02579	LMRG_02580	σ^B^	No	*htrA*, σ^A^-dependent expression pattern; σ^B^-dependent transcription generates a long (473 nt) 5′UTR, which may have regulatory function
24	LMRG_02624	–	σ^B^, σ^A^	No	*hly*, has a PrfA box, a σ^A^-dependent promoter, and a putative σ^B^-dependent promoter suggested in previous studies
25	LMRG_02632	–	σ^B^, σ^A^	Frags 8396 to 8402	*ldh*
26	LMRG_02731	–	σ^A^, σ^B^	No	–
27	LMRG_02772	–	σ^A^, σ^B^	Frags 68894 to 68895	–

a *Each fragment is composed of one or more 51 nt overlapping windows*.

### Some σ^B^-dependent TUs are also regulated by other transcriptional regulators

Three TUs previously reported as σ^B^-dependent, but not identified as such in our study reported here, were found to be co-regulated by σ^B^-dependent promoters and other alternative sigma factors and transcriptional factors. LMRG_02294, encoding a hypothetical protein, is co-transcribed with LMRG_02293, a gene encoding a putative amidohydrolase. A σ^B^-dependent−10 region and a σ^L^-dependent promoter in close proximity have been identified for this TU, suggesting co-regulation of transcription (Supplementary Table 6). As the strains used in this study lack an active σ^L^, overexpression of σ^B^ itself might not be sufficient to significantly upregulate the TU. LMRG_02294 only showed a non-significant upregulation in the strain expressing σ^B^.

LMRG_00906 (*rpoD*, encoding σ^A^), which is part of the LMRG_00911-LMRG_00906 TU, is co-transcribed from an upstream σ^B^-dependent promoter, a σ^H^-dependent promoter upstream of LMRG_00908 and a σ^A^-dependent promoter upstream of LMRG_00911. Strong expression from the σ^A^-dependent promoter as well as the absence of σ^H^ may explain why no significant differential expression of *rpoD* was observed in our study. LMRG_02624 (*hly*) has a PrfA box, a σ^A^-dependent promoter, and a putative σ^B^-dependent promoter suggested in previous studies (Orsi et al., 2008; Tsai et al., 2011). *hly* was only reported as σ^B^-dependent with active expression of PrfA (Ollinger et al., 2009; Toledo-Arana et al., 2009; Ribeiro et al., 2014), which was not shown to be significantly differentially expressed in our study.

## Discussion

A number of studies have analyzed *L. monocytogenes* alternative-sigma-factor-dependent expression profiles at the transcriptional and protein levels based on comparisons between WT and single deletion mutant strains. However, regulon redundancy and overlaps in regulation by multiple sigma factors is common, as has been shown in *Listeria* (Chaturongakul et al., 2011), *B. subtilis* (Mascher et al., 2007), and in other bacteria (Nuss et al., 2010). We thus used an experimental design that enabled us to study the functions and genes regulated by σ^B^ in the absence of all other alternative sigma factors in order to further explore the *L. monocytogenes* σ^B^ regulon and σ^B^-dependent gene regulation. Over-expression of a single alternative sigma factor in the absence of all other alternative sigma factors, the specific strategy used here, has previously been shown to eliminate redundant regulation by multiple alternative sigma factors, thus allowing improved insights into both the function of a single sigma factor and coregulation by different sigma factors (Mujahid et al., 2013a; Wang et al., 2014; Liu et al., 2016). As detailed above, this approach allowed us to (i) identify new σ^B^-dependent functions in *L. monocytogenes*, such as regulation of genes involved in 1,2-propanediol utilization and biosynthesis of pyrimidine nucleotides; (ii) identify new σ^B^-dependent genes involved in stress response and virulence functions; and (iii) further define a role for σ^B^ in *L. monocytogenes* homeostasis.

### *L. monocytogenes* σ^B^ directly upregulates >100 TUs and >200 genes

Our approach shows that the σ^B^ regulon includes >100 TUs and >200 genes that are directly up-regulated by σ^B^. As RNA-seq has been well documented, in both *L. monocytogenes* (Oliver et al., 2009) and other organisms (Vivancos et al., 2010), to provide quantitative data that are well correlated with qPCR data, qPCR confirmation of genes newly identified as σ^B^-dependent was not deemed necessary; rather σ^B^-dependent transcriptional start sites were confirmed for selected genes via 5′ RACE. By comparison, previous studies have reported the σ^B^ regulon in 10403S as including >110 genes and >80 promoters (Raengpradub et al., 2008; Abram et al., 2008a,b; Oliver et al., 2009; Ollinger et al., 2009; Chaturongakul et al., 2011). Combined results from previous microarray, RNA-seq and proteomic studies in different *L. monocytogenes* strains under different stress conditions show a large σ^B^ pan-regulon of up to 902 genes (Wemekamp-Kamphuis et al., 2004; Chatterjee et al., 2006; Abram et al., 2008a,b; Ollinger et al., 2008, 2009; Raengpradub et al., 2008; Oliver et al., 2009, 2010; Toledo-Arana et al., 2009; Chaturongakul et al., 2011; Ribeiro et al., 2014) and a core regulon in lineage II strains of 184 genes, as supported by three or more previous experiments. Overall, our data further confirm that σ^B^ is the alternative sigma factor that regulates the largest regulon; other regulators that directly regulate large gene sets in *L. monocytogenes* include σ^H^, which directly regulates more than 60 genes (Chaturongakul et al., 2011; Liu et al., 2016).

### Newly identified σ^B^-dependent promoters reveal a novel role for σ^B^ in oxidative stress, 1,2-propanediol utilization and biosynthesis of pyrimidine nucleotides

While *L. monocytogenes* σ^B^ clearly has been shown to be important for survival under many stress conditions, such as exposure to heat, bile, osmotic and acid stress (Ferreira et al., 2001, 2003; Sue et al., 2004; Seifart Gomes et al., 2011), the role of σ^B^ in oxidative stress response is less clear. Previous studies have reported results ranging from hypersensitivity (Ferreira et al., 2001; Oliver et al., 2010) to hyperresistance (Moorhead and Dykes, 2003; Boura et al., 2016) to oxidative stress for σ^B^ null mutants. For example, a previous study reported that viability of a 10403S Δ*sigB* mutant was 100-fold lower than the WT under oxidative stress induced by cumene hydroperoxide in stationary phase (Ferreira et al., 2001). However, a recent study using *L. monocytogenes* 10403S and EGD-e showed that deletion of *sigB* led to hyperresistance to H_2_O_2_ in stationary phase cells grown under aerobic conditions (Boura et al., 2016). Further, while studies in *B. cereus* showed that loss of σ^B^ or σ^B^ regulon members showed impaired glucose-starvation-induced resistance to H_2_O_2_ (Engelmann and Hecker, 1996; Antelmann et al., 1997; Zuber, 2009), the Δ*sigB* mutant showed either a similar degree of resistance (Engelmann and Hecker, 1996) or hyperresistance to H_2_O_2_ induced oxidative stress (Van Schaik et al., 2005). Interestingly, we found both *hslO* and the *qoxABCD* operon to be transcribed from a σ^B^-dependent promoter, suggesting a direct role of σ^B^ in resistance to oxidative stress. Among heat shock proteins, HslO has a unique chaperone activity because it is redox regulated and protects both thermally and oxidatively damaged proteins from irreversible aggregation (Kim et al., 2001). The importance of *qoxABCD* in oxidative stress response is also supported by observations in *B. subtilis* where a *qoxABCD* mutant shows reduced growth under aerobic conditions (Winstedt and Von Wachenfeldt, 2000). Upregulation of *qox* genes in *L. monocytogenes* in the presence of glycerol further indicates their involvement in adaption to an aerobic environment (Joseph et al., 2008). Our results suggest that the operon *qoxABCD* should be a focus for future studies on the molecular mechanisms behind oxidative stress resistance. As the exact role of σ^B^ in oxidative stress response in *L. monocytogenes* has not been elucidated to date, identification of these genes as regulated by σ^B^-dependent promoters may provide fresh insight into the role of σ^B^ in oxidative stress response.

Interestingly, our data also found the propanediol utilization (*pdu*) operon to be transcribed from an upstream σ^B^-dependent promoter, revealing a new role for σ^B^ in the utilization of 1,2-propanediol. 1,2-propanediol is produced during the catabolism of rhamnose and fucose, two sugars that are abundant in mammalian glycoconjugates (Staib and Fuchs, 2014). In addition, 1,2-propanediol is also used in various food products as a direct food additive (Cameron et al., 1998). The metabolism of 1,2-propanediol has been well studied in *Salmonella*; this organism is not only able to use 1,2-propanediol as a carbon source, but the *pdu* operon has also been shown to be important for virulence in *Salmonella* (Bobik et al., 1999). So far, studies in *L. monocytogenes* showed that transcription of some of the *pdu* genes increases under certain conditions, including lack of glucose (Nilsson et al., 2005), during intracellular growth (Joseph et al., 2006), and during growth on the surface of cold smoked salmon slices (Tang et al., 2015). In *L. innocua*, the *pduD* gene is required for 1,2-propanediol metabolism and 17 genes within the *pduA-*to-*pduF* gene cluster are induced when cells are grown in a medium containing 1,2-propanediol (Xue et al., 2008). The studies detailed above suggest the importance of 1,2-propanediol utilization in *Listeria* during growth in foods and in the host environment; our study reveals evidence for a regulatory role for σ^B^ in 1,2-propanediol utilization. This finding further illustrates important regulatory roles for σ^B^ in adaptation and transition to food and host environments.

Another σ^B^-dependent promoter newly identified here is upstream of the LMRG_00978 to LMRG_00986 operon, which encodes proteins involved in pyrimidine ribonucleotide biosynthesis. Previous studies showed that genes involved in the *de novo* synthesis of purines (*purA, purQ*, lmo1771) and pyrimidines (*pyrE*) are required for intracellular proliferation of *L. monocytogenes*, suggesting that these bases and nucleotides are not provided by the host cell, but must be synthesized by the bacterium (Schauer et al., 2010). Our study supports σ^B^ as one of the key regulators for nucleotide metabolism, which is essential for *L. monocytogenes* to succeed in the intracellular environment.

### Newly identified σ^B^-dependent promoters reveal additional mechanisms for σ^B^-dependent roles in osmotic stress, glycerol utilization, and virulence

In addition to identification of new σ^B^-dependent functions, as detailed above, our data also newly identified σ^B^-dependent genes involved in functions previously linked to σ^B^ regulation, including oxidative stress, osmotic stress, glycerol utilization, and virulence. The response to osmotic stress is one of the first reported specific functions for σ^B^ in *L. monocytogenes* (Becker et al., 1998). Specific genes involved in osmotic stress response, such as *opuCA*, have a confirmed σ^B^-dependent promoter (Kazmierczak et al., 2003) and are activated by osmotic stress in a σ^B^-dependent manner (Sue et al., 2004). The osmotic activation of σ^B^ in *L. monocytogenes* is rapid, transient, and proportional to the magnitude of the osmotic stress applied (Utratna et al., 2011). Here, two operons with a role in osmotic stress response were newly identified as being directly regulated by σ^B^, including (i) LMRG_02114-LMRG_02116 and (ii) LMRG_02775-LMRG_02778, which includes one gene encoding an OsmC/Ohr family protein. Previous studies have found that one of two genes encoding OsmC/Ohr family proteins is a member of the σ^B^ regulon in *B. subtilis* (Kempf and Bremer, 1995; Volker et al., 1998), suggesting a general role for σ^B^-dependent regulation of OsmC/Ohr family proteins. These osmotic stress response proteins, newly identified to be σ^B^-dependent, broaden our knowledge of how σ^B^ fine-tunes gene regulation to support chemical and water homeostasis in *L. monocytogenes*. We hypothesize that these σ^B^-dependent genes could play an important role under high osmotic stress conditions such as those encountered by *L. monocytogenes* during gastrointestinal passage (Sue et al., 2004).

Glycerol is used as an alternative carbon source by intracellular *L. monocytogenes* (Eylert et al., 2008). Previous publications also suggested the importance of the ability to metabolize a series of carbon sources for successful infection by intracellular human pathogens (Olive and Sassetti, 2016) such as *M. tuberculosis* (de Carvalho et al., 2010). In *L. monocytogenes*, the mutant defective in the uptake and metabolism of glycerol showed impaired intracellular growth (Eylert et al., 2008). In a previous proteomics study, σ^B^ was found to regulate the LMRG_02000-LMRG_02002 operon, which encodes subunits of dihydroxyacetone kinase, an enzyme involved in glycerol metabolism in *L. monocytogenes* (Abram et al., 2008b). In addition, a *sigB* mutant had a diminished ability to use glycerol as a sole carbon source (Abram et al., 2008b). In our study, the LMRG_01432-LMRG_01431 operon, which encodes a glycerol kinase and a glycerol uptake facilitator protein, was found to have a σ^B^-dependent promoter, further supporting an important role for σ^B^ in regulation of glycerol metabolism. As growth in glycerol also upregulates PrfA activity (Joseph et al., 2008; Stoll et al., 2008), this newly revealed role for σ^B^ in glycerol metabolism further supports a link between carbohydrate metabolism and pathogenesis in *L. monocytogenes*.

While σ^B^ previously has been recognized as a transcriptional regulator of a number of virulence genes, including *prfA, inlA, inlC2*, and *inlD* (Mcgann et al., 2007; Guldimann et al., 2016), our study revealed that another virulence gene, *srtA*, which is involved in proteolysis and processing of internalin proteins (Garandeau et al., 2002), is also directly regulated by σ^B^. SrtA is required for the cell wall anchoring of InlA and, presumably, for the anchoring of other LPXTG-containing proteins that are involved in listerial infections (Bierne et al., 2002). A recent study also reported that inhibition of sortase A by chalcone could prevent *L. monocytogenes* infection (Li et al., 2016). In *S. aureus*, a number of sortase substrate proteins were previously observed to have higher or lower expression in a the Δ*sigB* mutant (Hempel et al., 2010). Our finding confirms and extends the role σ^B^ plays in pathogenesis, especially in the interaction between pathogen and host surface molecules during host cell invasion.

### Co-regulation of genes by σ^B^ and σ^A^ as well as other transcriptional regulators supports importance of σ^B^ in resilience and homeostasis

While *L. monocytogenes* σ^B^ has previously been well defined as a regulator of stress response and virulence functions, our data reported here further support a broader role for σ^B^ in resilience and homeostasis. The enriched GO terms related to general transport and homeostasis confirmed contributions of σ^B^ in coordinating complex networks responsive to changing environmental conditions.

Importantly, we also found a number of σ^B^-dependent TUs and genes preceded by both σ^B^ and σ^A^-dependent promoters (Supplementary Table 7). Key genes in this category include *prfA, rsbV, qoxABCD* (aerobic respiration), *mogR* (repressor of genes involved in flagella)*, phoU* (phosphate transport regulation)*, cggR* (central glycolytic genes regulator)*, ltrC* (response to cold) and others. Genes preceded by both a promoter regulated by a housekeeping sigma factor and a promoter regulated by an alternative sigma factor have been identified in other bacteria. For example, in *B. subtilis, clpC* is preceded by two promoters (σ^A^ and σ^B^) (Kruger et al., 1996) and expression of the *ureABC* operon, which encodes urease, is dependent on σ^A^ and σ^H^ (Wray et al., 1997). Additionally, the Extracytoplasmic Function (ECF) sigma factors of *B. subtilis* are known to co-regulate promoter regions with an existing σ^A^ promoter (Eiamphungporn and Helmann, 2008; Kingston et al., 2011). In *E. coli*, extensive overlaps between promoters of the primary sigma factor σ^70^ and alternative sigma factors such as σ^32^ and others also suggest coregulation of gene expression by multiple sigma factors under various growth conditions (Wade et al., 2006).

Seventy-four genes identified in previous studies as σ^B^-dependent were not differentially expressed as σ^B^-dependent in this study. A considerable number of these genes were co-regulated by more than one sigma factor, predominantly by σ^B^ and σ^A^. A likely explanation regarding why some previously identified σ^B^-dependent genes showed no differential expression in this study may reflect the growth conditions under which the experiments were conducted: the cells were in logarithmic growth with no additional imposed stress conditions. Previous studies used stress conditions such as stationary phase and salt stress to induce σ^B^ expression. In contrast, our experiment used artificial induction of σ^B^ expression by addition of rhamnose to log-phase cells. The transcriptional activity of the σ^A^ housekeeping sigma factor has been found to be diminished under stress conditions (Sharma and Chatterji, 2010; Delumeau et al., 2011). Fast-growing cells depend on σ^A^ to regulate expression of housekeeping genes such as those involved in protein biosynthesis, DNA replication and structural proteins. Therefore, under log-phase, σ^A^ is usually highly active to support the reproductive needs of the cells (Delumeau et al., 2011). Under the conditions used in this study, genes co-transcribed by both σ^A^ and σ^B^ under log-phase might have very little expression originating from the sometimes weaker σ^B^ promoter if the stronger σ^A^ promoter is located upstream or very close to the σ^B^ promoter.

## Conclusions

In this study, we used RNA-seq to explore the role of σ^B^ in *L. monocytogenes* by overexpressing σ^B^ in a strain where genes encoding all other alternative sigma factors had been deleted. Combined with prior data revealing important roles for σ^B^ in pathogenesis and stress response, identification of new putative σ^B^-dependent promoters upstream of a number of genes indicates a broader regulon for this alternative sigma factor, which also appears to contribute to cellular homeostasis.

Transcriptomic approaches such as RNA-seq and microarrays as well as proteomic approaches are powerful tools to explore the regulation of sigma factors, however, they have limited abilities to distinguish indirectly regulated genes from directly regulated genes. Further experiments with approaches such as ChIP-seq may allow a better definition of the direct regulons of sigma factors.

## Author contributions

YL performed the RNA-seq experiments and initial data analyses and was a major contributor in writing the manuscript; RO performed sliding window analysis of RNA-Seq data and promoter identification; KB, MW, and VG co-wrote the manuscript and conceived the study. All authors read and approved the final manuscript.

### Conflict of interest statement

The authors declare that the research was conducted in the absence of any commercial or financial relationships that could be construed as a potential conflict of interest.

## Supplementary material

Supplementary Figure 1 qRT-PCR results of rhamnose induction of *sigB* and the σ^B^-dependent gene LMRG_01602. Expression of σ^B^ under rhamnose induction was tested by qRT-PCR. LMRG_01602 was used as the targeted σ^B^-dependent gene and *rpoB* as the reference gene; error bars show the standard deviation for the 2 biological replicates; Y axis represents the fold change of the gene expression level. Fold change is calculated as −ΔCt = Ct_rpoB_ − Ct_target gene_. The σ^B^-dependent gene referred in second graph is LMRG_01602.

Supplementary Table 1 Plasmid, primers and strains used in this study.

Supplementary Table 2 Genes and TUs upregulated by σ^B^ identified by traditional BaySeq and sliding window analyses.

Supplementary Table 3 Genes downregulated by σ^B^ identified by traditional BaySeq.

Supplementary Table 4 σ^B^-dependent TUs identified by sliding window analyses, but not by the traditional BaySeq approach.

Supplementary Table 5 List of NDE σ^B^-dependent genes.

Supplementary Table 6 5′UTRs of the NDE σ^B^-dependent genes.

Supplementary Table 7 TUs with σ^B^-dependent promoters.

## Abbreviations

asRNA: anti-sense RNA
BHI: Brain Heart Infusion
BWA: Burrows-Wheeler Aligner
DE: differentially expressed
ECF: Extracytoplasmic Function
FC: fold change
FDR: false discovery rate
GEO: Gene Expression Omnibus
GO: Gene Ontology
ncRNA: noncoding RNA
NDE: not differentially expressed
NEB: New England Biolabs
NIAID: National Institute of Allergy and Infectious Diseases
NIH: National Institutes of Health
NRC: normalized RNA-seq coverage
ORF: open reading frame
5′ RACE: 5′ Rapid amplification of cDNA ends
RNA-seq: RNA sequencing
TU: transcription unit
UTR: untranslated region
WT: wild type
TSS: transcription start sites.
