# Supplementary material for: Home Alone: Elimination of All but One Alternative Sigma Factor in Listeria monocytogenes Allows Prediction of New Roles for σB
Source: Front Microbiol. 2017 Oct 11;8:1910. doi: 10.3389/fmicb.2017.01910 (PMC5641562; doi:10.3389/fmicb.2017.01910)

Supp. Figure 1. qRT-PCR results show that under 50mM Rhamnose induction, σ^B is overexpressed and the σ^B-dependent gene is also upregulated

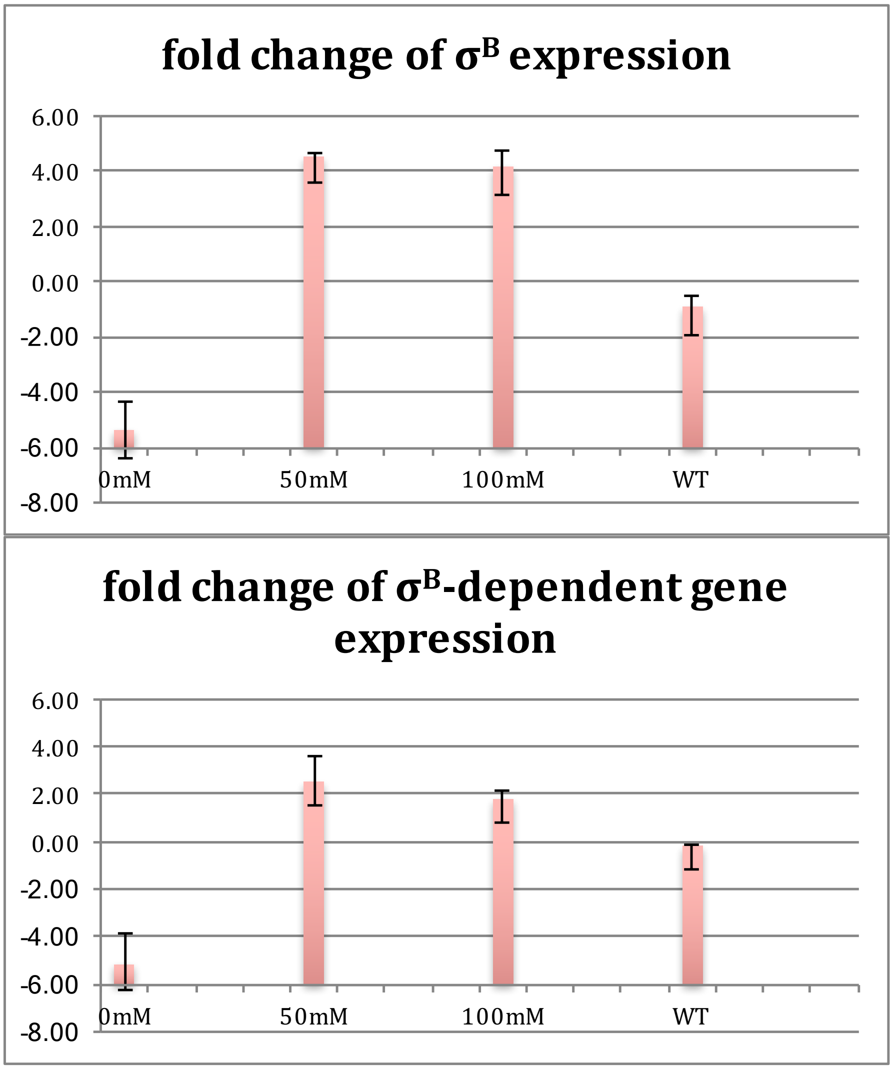

Supplement: Supplementary Figure 1 — qRT-PCR results of rhamnose induction of sigB and the σB-dependent gene LMRG_01602. Expression of σB under rhamnose induction was tested by qRT-PCR. LMRG_01602 was used as the targeted σB-dependent gene and rpoB as the reference gene; error bars show the standard deviation for the 2 biological replicates; Y axis represents the fold change of the gene expression level. Fold change is calculated as −ΔCt = CtrpoB − Cttarget gene. The σB-dependent gene referred in second graph is LMRG_01602. [file Image1.PDF]
